# Navigating the complexities of dual CNV findings: a case of DEE7 caused by a *de novo KCNQ2* deletion and a Co-occurring chromosome 13 duplication – a case report and literature review

**DOI:** 10.3389/fped.2026.1737877

**Published:** 2026-05-14

**Authors:** Yangfan Qi, Shuangzhu Lin, Ying Zhou, Yanzhi Chen, Xiaochun Feng, Kai Jiang, Jinhua Feng

**Affiliations:** 1 Changchun University of Chinese Medicine, Changchun, China; 2 Diagnosis and Treatment Center for Children, The Affiliated Hospital of Changchun University of Chinese Medicine, Changchun, China; 3 Norman Bethune College of Medicine, Jilin University, Changchun, China

**Keywords:** copy number variation, developmental and epileptic encephalopathy 7, gene deletion, *KCNQ2*, neonatal epilepsy, whole exome sequencing

## Abstract

**Background:**

Developmental and epileptic encephalopathy 7 (DEE7) is a severe neurodevelopmental disorder stemming from mutations in the *KCNQ2* gene, which encodes a critical voltage-gated potassium channel subunit. While *KCNQ2* mutations are known to cause a spectrum of diseases, from benign familial neonatal seizures (BFNS1) to drug-resistant DEE7, the clinical outcomes specifically associated with large deletions—a rare mechanism causing haploinsufficiency—are not well defined. This study reports a *de novo KCNQ2* large deletion and aims to delineate the associated clinical trajectory and treatment response.

**Case summary:**

We report a 1 year 5 months female who presented with frequent seizures starting in the neonatal period (7 days after birth), characterized by limb stiffness and facial cyanosis, with a frequency of 4–5 episodes per week. Initial neurological examination revealed no focal deficits, and brain MRI showed no structural abnormalities. However, electroencephalography demonstrated slowed background activity with multifocal epileptiform discharges. Standardized neurodevelopmental assessments confirmed severe global delay (Griffiths Developmental Quotient of 60 and Alberta Infant Motor Scale score below the 5th percentile at 3 months of age). Trio-based whole-exome sequencing was performed, identifying a ∼1.62 Mb *de novo* heterozygous deletion at chromosome 20q13.33 and a co-occurring ∼4.68 Mb duplication at chromosome 13q34 in the proband. This variant was classified as pathogenic according to American College of Medical Genetics and Genomics (ACMG) guidelines and fully encompassed the *KCNQ2* gene, leading to a definitive diagnosis of *KCNQ2*-related developmental and epileptic encephalopathy 7 (DEE7).

**Conclusion:**

We diagnosed DEE7 caused by a large *KCNQ2* deletion using trio whole-exome sequencing and multiplex ligation-dependent probe amplification (MLPA). Despite the severe genotype, early targeted treatment led to complete seizure freedom and modest neurodevelopmental progress. This case demonstrates that seizure freedom and some developmental gains are achievable, highlighting the potential benefit of early intervention even in patients with severe genotypes and underscoring the need for copy number variation (CNV) analysis in epilepsy genetic testing.

## 1 Introduction

DEE7 ^(OMIM #613720)^ is a severe neurological disorder characterized by refractory seizures in early infancy and global developmental delay. The electroencephalogram (EEG) initially may demonstrate a burst-suppression pattern, which may later evolve into multifocal epileptiform activity. Neuroimaging in some patients reveals abnormalities of the basal ganglia (1). With advances in diagnostic techniques, an increasing number of monogenic etiologies have been identified, among which mutations in the potassium voltage-gated channel subfamily Q member 2 (*KCNQ2*) gene represent one of the important causes of DEE7.

The *KCNQ2* gene ^(OMIM #602235)^, located on chromosome 20q13.33, encodes the Kv7.2 potassium channel protein, a key component of the neuronal M-channel. The M-channel plays a critical role in regulating neuronal excitability and synaptic transmission. Loss of function in *KCNQ2* leads to neuronal depolarization and increased excitability, thereby triggering seizures. Mutations in *KCNQ2* cause neonatal-onset epileptic disorders with marked phenotypic heterogeneity, spanning a clinical spectrum from milder forms such as benign familial neonatal seizures (BFNS) to severe phenotypes like developmental and epileptic encephalopathy 7 (DEE7) (2, 3).

In *KCNQ2*-related developmental and epileptic encephalopathy 7 (DEE7), the majority of reported cases are caused by missense mutations (4). In contrast, large deletions of the *KCNQ2* gene leading to haploinsufficiency are relatively uncommon. A limited number of studies have suggested that such deletions are typically associated with severe phenotypic manifestations (5). Nevertheless, comprehensive characterization of long-term treatment responses and neurodevelopmental trajectories in these patients remains scarce, and whether phenotypic heterogeneity exists in their prognosis is still not well defined.

Therefor,this study identified a case of DEE7 caused by a *de novo* large *KCNQ2* deletion, accompanied by a chromosome 13q34 duplication, through integrated trio whole-exome sequencing and copy number variation (CNV) analysis. The patient presented with severe neonatal seizures and developmental delay but achieved complete seizure control and significant neurodevelopmental catch-up following early combined pharmacological intervention. By elucidating this distinctive clinical trajectory, our work aims to explore prognostic diversity in such genotypes and provide new evidence supporting early precision diagnosis, active intervention, and the essential role of CNV analysis.

## 2 Case presentation

The patient first exhibited seizures on the seventh day of life without an obvious trigger. The initial episode involved loss of consciousness, fixed upward gaze, generalised rigidity, cyanosis of the face and lips, and deviation of the mouth angle, without drooling or incontinence. The event lasted approximately one minute and resolved spontaneously, followed by postictal somnolence. Subsequently, the seizures recurred in various forms, including: 1) sudden startles during sleep followed by intense crying and generalised stiffness, lasting 1–3 min; 2) upward gaze, neck extension, and increased limb tone while awake, lasting around 20 s; and 3) episodes provoked by auditory or tactile stimuli, manifesting as pallor, fixed stare, and limb rigidity, lasting 10–30 s. These events occurred frequently, with a frequency of 3–5 episodes per week, leading to multiple hospital admissions at the First Hospital of Jilin University.

Physical Examination:Her length was 76 cm (∼25th percentile), weight 9.5 kg (25th–50th percentile), and head circumference 46 cm (∼50th percentile). She was well-developed, alert, and in good spirits. No cranial deformities were noted. Ocular movements were normal, with prompt pupillary reaction to light and good visual tracking. The skin was clear without rash or café-au-lait spots. Face, neck, trunk, and limbs were unremarkable. Cardiorespiratory and abdominal examinations revealed no abnormalities.Notably, the neurological examination showed no focal deficits: physiological reflexes were present, pathological reflexes were absent, and muscle strength and tone were normal in all four limbs.

Birth and Family History:The infant was the first child of her mother (G1P1), delivered by caesarean section at 38 weeks of gestation. Her birth length was 48 cm (approximately 15th–25th centile) and birth weight was 2.8 kg (approximately 10th–15th centile). There was no history of perinatal asphyxia or complications. The mother had a history of chronic hypertension prior to pregnancy. During gestation, her blood pressure was managed with nifedipine and labetalol, with satisfactory control. She reported a personal history of febrile seizures during early childhood. There were no other family members with seizure disorders, and no further significant family history of inherited conditions.

### 2.1 Clinical workup

A comprehensive evaluation—including full blood count, urinalysis, stool analysis, liver and renal function tests, cardiac enzymes, electrolyte panel, thyroid function, and genetic metabolic screening in blood and urine—revealed no abnormalities. Brain MRI was reported as follows: Symmetrically distributed signals in the periventricular white matter, considered consistent with the myelination process;Widened bilateral temporo-occipital subarachnoid spaces.

Electroencephalography (EEG) studies showed evolving features: At 13 days of age: Background activity of 3–10 Hz, consistent with a mildly abnormal neonatal EEG pattern, featuring delayed cerebral maturation and multifocal sharp waves, spike waves, and polyspike-wave discharges. At 17 months of age: A well-organised occipital dominant rhythm of 5–6 Hz theta (low-to-moderate amplitude) was noted, bilaterally symmetrical. This was interpreted as a normal EEG.

Baseline Neuro developmental Assessments Following the initial diagnosis, the infant underwent serial standardised developmental evaluations. (Tables 1 and 2).

**Table 1:** Longitudinal developmental profile of the patient on the griffiths mental development scales.

Assessment Scale and Age	Domain	Developmental Quotient (DQ)	Age Equivalent (months)
Griffiths (3m 13d)	Locomotor	43	1.5
	Personal-Social	71	2.5
	Hearing and Speech	86	3.0
	Eye-Hand Coordination	29	1.0
	Performance	71	2.5
	General Quotient (GQ)	60	
Griffiths (12m 12d)	Locomotor	80	10.0
	Personal-Social	52	6.5
	Hearing and Speech	52	6.5
	Eye-Hand Coordination	60	7.5
	Performance	52	6.5
	General Quotient (GQ)	59	
Griffiths (17m)	Locomotor	68	11.5
	Personal-Social	71	12.0
	Hearing and Speech	44	7.5
	Eye-Hand Coordination	68	11.5
	Performance	44	7.5
	General Quotient (GQ)	69	

AIMS, alberta infant motor scale; DQ, developmental quotient.

**Table 2:** Serial assessments with the Alberta infant motor scale (AIMS).

Assessment Age	AIMS Score	Percentile	Interpretation
3m 13d	7	< 5th	5Significant motor delay
12m 12d	45	< 5th	Persistent significant motor delay
17m	54	< 5th	Motor delay persists, but raw score shows substantial improvement

### 2.2 Clinical diagnostic reasoning

Given the early-onset refractory seizures and severe global developmental delay, coupled with normal routine metabolic screening and neuroimaging, a genetic etiology was highly suspected. Therefore, whole-exome sequencing (WES) was ordered to clarify the underlying cause.

### 2.3 Genetic analysis

**Figure 1:**
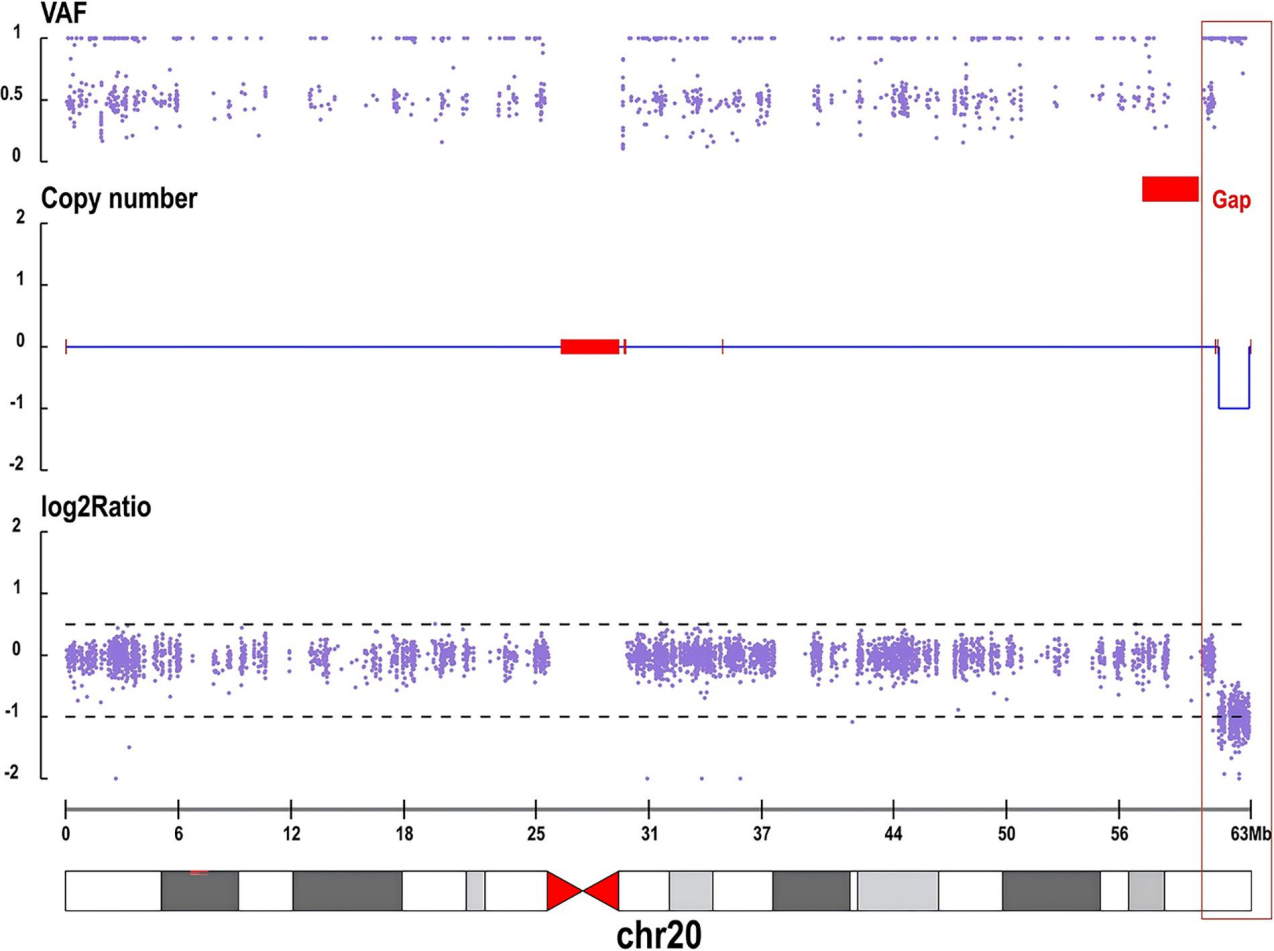
Copy number analysis of chromosome 20 in the proband, revealing a heterozygous deletion encompassing the *KCNQ2* gene.the red box highlights an approximately 1.62 Mb heterozygous deletion at chromosomal region 20q13.33 (genomic coordinates: chr20:61,000,000-63,000,000). This deletion is characterized by a loss of heterozygosity in the B-allele frequency (VAF) plot, a copy number reduction to 1, and a significant decrease in the log2 ratio. The deleted region fully encompasses the *KCNQ2* gene.

To further investigate the aetiology, and following informed consent from the guardians, trio whole-exome sequencing was performed on the proband and her parents. Bioinformatic analysis included simultaneous investigation of single-nucleotide variants and small insertions/deletions, as well as copy number variant analysis based on sequencing depth. The analysis identified two copy number variants in the proband: firstly, a ∼4.68 Mb duplication of uncertain significance at chromosome 13q34, and secondly, a *de novo* ∼1.62 Mb heterozygous deletion at chromosome 20q13.33. Both parents were wild-type at this latter locus. According to the American College of Medical Genetics and Genomics guidelines, the chromosome 13 duplication was classified as a variant of uncertain significance (1A + 3A + 4C + 5A, total score 0.25), whereas the chromosome 20 deletion was classified as pathogenic (1A + 2A + 3C + 5A, total score 2.05) (Figure 1). Notably, the deleted region on chromosome 20 fully encompasses the KCNQ2 gene, for which haploinsufficiency is an established disease mechanism.

Final diagnosis: developmental and epileptic encephalopathy 7.

### 2.4 Treatment

Upon diagnosis, an individualised treatment plan was formulated for the patient, with adjustments made based on therapeutic response.Initially, at one month of age, she was started on levetiracetam monotherapy (30 mg/kg/day) supplemented with vitamin B₆ (three tablets twice daily). Due to inadequate seizure control over the following fortnight, the dose of levetiracetam was gradually increased to 50 mg/kg/day by day 45 of life, and further to 55 mg/kg/day three days later. At that point, oxcarbazepine was introduced at a starting dose of 1.5 mL twice daily. Following this adjustment, the frequency, duration, and intensity of seizures decreased markedly, with complete seizure freedom achieved by six months of age. After maintaining this status for over nine months, the regimen was gradually simplified. Levetiracetam was successfully tapered and discontinued. Currently, at 17 months of age, the patient remains seizure-free on oxcarbazepine monotherapy (2 mL twice daily), supplemented with vitamin B₆ (two tablets once daily) and vitamin D (10,000 units once weekly). Her growth and development have been satisfactory.

### 2.5 Outcome and follow-up

The patient has been followed up longitudinally, and as of 17 months of age, she has shown marked clinical improvement, primarily evidenced by complete seizure freedom and gains in motor and social domains, although global neurodevelopmental delay persists. (1) Growth: Her height was 76 cm (∼25th centile), weight 9.5 kg (25th–50th centile), and head circumference 44 cm (∼3rd centile). Anthropometric assessment indicated proportionate growth in height and weight, however, head growth has been relatively delayed, warranting continued monitoring. (2) Seizure Outcome: The patient attained complete seizure freedom from 5 months of age, which has now been maintained for over 12 months. (3) Medication: Following sustained seizure freedom, levetiracetam was tapered and discontinued at 14 months of age. She currently remains seizure-free on oxcarbazepine monotherapy (2 mL twice daily). (4) Neurodevelopment: At 17 months, the overall Griffiths developmental quotient (DQ) was 69, consistent with mild global delay. Her developmental profile is notably uneven: personal–social (DQ 71) and motor (DQ 68) skills have shown clear catch-up, approaching the 12-month level, while hearing–speech (DQ 44) and performance (DQ 44) domains remain significantly delayed, equivalent to a 7.5-month level. In addition, the Alberta Infant Motor Scale (AIMS) score (<5th centile) confirms that her gross motor abilities, despite improvement, remain well below those of peers.

## 3 Discussion

The *KCNQ2* gene is a well-established cause of developmental and epileptic encephalopathy (DEE), with a broad mutational spectrum and diverse clinical presentations. Genotype-phenotype correlations indicate that the type of mutation is a key prognostic factor. While the majority of reported cases are caused by point mutations, which exhibit considerable heterogeneity in clinical severity and outcome—some missense mutations even leading to relatively mild phenotypes or benign familial neonatal seizures—large deletions resulting in haploinsufficiency are less common. These deletions, causing a complete loss of one Kv7.2 channel protein copy, are generally associated with more severe neurodevelopmental outcomes in the literature (5). The pathogenic mechanism involves the complete loss of *KCNQ2* function, which severely disrupts neuronal M-channel activity, leading to uncontrolled hyperexcitability in neuronal networks and, consequently, earlier, more difficult-to-control seizures and a more severe encephalopathy. Against this backdrop, we report a case with a uniquely favorable trajectory, providing new evidence for a deeper understanding of the prognosis and optimized management strategies for this genotype.

The early clinical presentation of our patient appeared “atypical” and somewhat inconsistent with the expected severity of her genotype. Although she experienced recurrent neonatal seizures, her neurological examination revealed no focal deficits, with normal muscle strength and tone. More importantly, her brain MRI showed no structural abnormalities, and her EEG, while demonstrating epileptiform discharges, lacked a typical burst-suppression pattern. This relatively “benign” initial presentation created a notable clinical paradox when contrasted with the “severe” genetic etiology of a large *KCNQ2* deletion. This very discrepancy prompted us to investigate the underlying cause through trio-based whole-exome sequencing.

Following informed consent, trio-WES and CNV analysis were performed. The analysis revealed two copy number variants in the proband: a *de novo* ∼1.62 Mb heterozygous deletion at chromosome 20q13.33 and a ∼4.68 Mb duplication of uncertain significance at chromosome 13q34. We systematically evaluated and interpreted the pathogenicity of these two variants according to ACMG guidelines. The deletion on chromosome 20 was classified as “pathogenic” because it was *de novo*, fully encompassed the established disease gene *KCNQ2*, and the gene's haploinsufficiency mechanism was highly consistent with the phenotype of DEE. In contrast, the duplication on chromosome 13, also *de novo*, was classified as a “variant of uncertain significance” (VUS) due to the lack of established dosage-sensitive genes in the region and weak phenotypic correlation with the patient's core epileptic symptoms.However, considering the patient's highly uneven neurodevelopmental profile—where motor skills showed significant catch-up but hearing and speech domains remained severely delayed (DQ 44)—this VUS warrants closer clinical attention. As suggested by the literature, distal 13q duplications can be associated with hearing impairment and neurodevelopmental delay (6). Therefore, it is highly plausible that the co-occurring 13q34 duplication played an additional pathogenic role, specifically contributing to her profound hearing-language deficits rather than being completely benign. Unfortunately, a formal objective audiometric evaluation was not performed during the current follow-up period, which represents a limitation of our study. Future follow-up for this patient will strictly necessitate comprehensive hearing assessments to further clarify the phenotypic impact of this structural variation.

Conversely, the ∼4.68 Mb duplication at 13q34 encompasses multiple genes, including *COL4A1*, *COL4A2*, and *ATP11A*. We performed a dosage-sensitivity analysis of these genes using public databases (e.g., ClinGen). While pathogenic variants in *COL4A1/2* are typically associated with structural brain anomalies not observed in our patient (7), the *ATP11A* gene is notably implicated in autosomal dominant non-syndromic hearing loss (DFNA33) (6). This molecular evidence, when aligned with the patient's clinical profile of profound hearing and language impairment, suggests that while *KCNQ2* remains the primary driver of epilepsy, the 13q34 duplication may synergistically contribute to the patient's complex systemic neurodevelopmental phenotype through a cumulative gene-dosage effect.

Ultimately, based on the clinical phenotype of infantile-onset epilepsy, EEG abnormalities, and developmental delay, coupled with the key molecular evidence of a pathogenic *KCNQ2* deletion, the patient was definitively diagnosed with *KCNQ2*-related developmental and epileptic encephalopathy 7.

Based on the definitive diagnosis of a *KCNQ2* deletion, we were able to implement a precise, combined anti-seizure treatment plan early in the disease course. In recent years, with a deeper understanding of the pathological mechanisms of *KCNQ2* channelopathies, sodium channel blockers (SCBs) such as oxcarbazepine/carbamazepine have been recognized as cornerstone therapies for *KCNQ2*-DEE7, demonstrating efficacy in controlling seizures and improving neurodevelopmental outcomes (8, 9). Their mechanism involves inhibiting voltage-gated sodium channels to reduce neuronal excitability, thereby indirectly compensating for the hyperexcitability caused by *KCNQ2* loss of function (10). Furthermore, other antiseizure medications like levetiracetam and topiramate have shown varying degrees of efficacy in combination therapy (11, 12), suggesting that a multi-mechanistic approach may be a rational strategy for optimizing outcomes. In the present case, the early initiation of a combination of oxcarbazepine and levetiracetam led not only to complete seizure freedom but also to notable developmental progress. The management of this case can be considered a success primarily in terms of seizure control and gains in specific neurodevelopmental domains. The patient achieved sustained seizure freedom, and her Griffiths scales showed clear catch-up in locomotor and personal-social skills, approaching the 12-month level. However, it is crucial to objectively acknowledge that comprehensive neurodevelopmental challenges persist. The patient's overall developmental quotient (DQ) of 69 at 17 months of age still indicates mild global delay, with severe lag in hearing-speech and performance domains, equivalent to a 7.5-month level. This underscores the necessity for future rehabilitation focus and long-term support.

In summary, utilizing an integrated diagnostic strategy of trio-WES and CNV analysis, we diagnosed a rare case of DEE7 caused by a *de novo* large heterozygous deletion of the *KCNQ2* gene. Although the infant presented with severe epilepsy and developmental delay in early infancy, early combined anti-seizure therapy resulted in complete seizure freedom and modest neurodevelopmental progress. This case of successful management, based on precise genetic diagnosis, demonstrates that in patients with genotypes typically associated with poor outcomes, such as large *KCNQ2* deletions, active, individualized, and early multi-drug intervention—coupled with close monitoring—can potentially create a critical window for improving seizure outcomes and fostering development in some domains, thereby potentially altering the disease's severe natural history. Our experience strongly underscores that incorporating comprehensive genomic techniques (such as WES integrated with CNV analysis) into the routine etiological diagnosis of DEE is an indispensable prerequisite for accurate prognostic assessment and effective clinical management.

## 4 Patient perspective

The patient’s legal guardian provided written informed consent for the publication of this case report.

## Data Availability

The original contributions presented in the study are included in the article/Supplementary Material, further inquiries can be directed to the corresponding author.
